# *Schistosoma mansoni* venom allergen-like protein 4 (SmVAL4) is a novel lipid-binding SCP/TAPS protein that lacks the prototypical CAP motifs

**DOI:** 10.1107/S1399004714013315

**Published:** 2014-07-25

**Authors:** Alan Kelleher, Rabih Darwiche, Wanderson C. Rezende, Leonardo P. Farias, Luciana C. C. Leite, Roger Schneiter, Oluwatoyin A. Asojo

**Affiliations:** a National School of Tropical Medicine, Baylor College of Medicine, Houston, TX 77030, USA; b Division of Biochemistry, Department of Biology, University of Fribourg, Chemin du Musée 10, CH 1700 Fribourg, Switzerland; c Centro de Biotecnologia, Instituto Butantan, São Paulo, SP, Brazil

**Keywords:** venom allergen-like protein, Ancylostoma secreted protein, *Schistosoma mansoni*, sperm-coating protein, TAPs, CAP, venom antigen 5, *Saccharomyces cerevisiae*, sterol binding

## Abstract

Schistosomiasis is a parasitic disease that affects over 200 million people. Vaccine candidates have been identified, including *Schistosoma mansoni* venom allergen-like proteins (SmVALs) from the SCP/TAPS (sperm-coating protein/Tpx/antigen 5/pathogenesis related-1/Sc7) superfamily. The first SmVAL structure, SmVAL4, was refined to a resolution limit of 2.16 Å. SmVAL4 has a unique structure that could not be predicted from homologous structures, with longer loops and an unusual C-terminal extension. SmVAL4 has the characteristic α/β-sandwich and central SCP/TAPS cavity. Furthermore, SmVAL4 has only one of the signature CAP cavity tetrad amino-acid residues and is missing the histidines that coordinate divalent cations such as Zn^2+^ in other SCP/TAPS proteins. SmVAL4 has a cavity between α-helices 1 and 4 that was observed to bind lipids in tablysin-15, suggesting the ability to bind lipids. Subsequently, SmVAL4 was shown to bind cholesterol *in vitro*. Additionally, SmVAL4 was shown to complement the *in vivo* sterol-export phenotype of yeast mutants lacking their endogenous CAP proteins. Expression of SmVAL4 in yeast cells lacking endogenous CAP function restores the block in sterol export. These studies suggest an evolutionarily conserved lipid-binding function shared by CAP proteins such as SmVAL4 and yeast CAP proteins such as Pry1.

## Abstract 2

The first structure of an *S. mansoni* venom allergen-like protein is presented.

## 1. Introduction   

It is estimated that over 200 million people in 74 countries in the tropics and subtropics suffer from the chronic neglected disease schitosomiasis or bilharzia, while an additional 600 million people are at risk (World Health Organization, 2002 ▶). The majority of these infections are caused by three species, *Schistosoma mansoni*, *S. hematobium* and *S. japonicum*, which live in the blood vessels. Schistosomiasis is transmitted by contact with water containing the infective larval stage, cercariae. The cercariae penetrate the skin and mature into the adult worms, which produce eggs that are excreted into the water supply. The miracidia that hatch from the eggs infect the intermediate hosts, *Biomphalaria* snails in the case of *S. mansoni*, within which they mature into cercariae. The snails release these infective cercariae into the water supply and the infection cycle continues (Farias *et al.*, 2012 ▶). In addition to the direct morbidity owing to schistosomiasis, the parasites wreak havoc on the body and have been associated with increasing the risk of urogenital, liver and bladder cancers (Khaled, 2013 ▶). The current drug of choice for treating schistosomiasis is praziquantel, but unfortunately the parasite is increasingly becoming resistant to this drug (Doenhoff *et al.*, 2002 ▶). Thus, the development of other therapeutic approaches, including vaccines, for schistosomiasis is under way.

The initial selection of experimental vaccine candidates was based on Gene Ontology functions from the *S. mansoni* genome, which identified putative surface-exposed/secreted proteins capable of modulatory roles against the host immune system (Verjovski-Almeida *et al.*, 2003 ▶). The experimental vaccine candidates include proteins belonging to the superfamily that is known as both the CAP (cysteine-rich secretory protein/antigen 5/pathogenesis related-1) and the SCP/TAPS (sperm-coating protein/Tpx/antigen 5/pathogenesis related-1/Sc7) superfamily. The diverse members of the CAP superfamily include insect and reptile venom allergens; thus, the *S. mansoni* orthologs were named *S. mansoni* venom allergen-like proteins (SmVALs; Chalmers *et al.*, 2008 ▶). The 28 SmVALs belong to two distinct subfamilies (group 1 and group 2) and the expression of these proteins is developmentally regulated (Chalmers *et al.*, 2008 ▶). Group 1 SmVALs are produced during parasitism and have been implicated to participate in host–parasite interactions. The members of group 1 can be considered to be orthologs of the major proteins secreted by infective L3 hookworms upon host entry (Goud *et al.*, 2005 ▶; Chalmers *et al.*, 2008 ▶). Group 1 is by far the larger group and its members include SmVAL1–5, SmVAL7–10, SmVAL12, SmVAL14, SmVAL15 and SmVAL18–29. Life-cycle expression profiles of some of the SmVALs has been obtained: specifically, SmVAL1, SmVAL4 and SmVAL10 are linked to host invasion, with SmVAL4 being expressed in both the cercariae and 3 h schistosomula (Chalmers *et al.*, 2008 ▶). SmVAL4 is secreted at stages linked to invasion, which makes it a viable choice as a vaccine candidate.

The rationale behind selecting secreted molecules as vaccines candidates in helminths is to interfere with parasite migration by blocking or impairing key processes, for example skin penetration, blood-vessel penetration and blood feeding. This rationale is being applied to the helminth *Necator americanus* by targeting two enzymes (Na-APR-1 and Na-GST-1) secreted by the gut of the hookworm, aiming to starve the organism (Hotez *et al.*, 2013 ▶). In schistosomiasis, cercarial proteins released into the skin should be the first proteins to be accessible to the immune system and thus could be considered to be viable vaccine candidates. Larval secretions are also highly immunogenic vaccine targets, as passive immunization with antisera to these secretions confers around 50% protection against challenge infection (Hewitson *et al.*, 2009 ▶). As a rationale for designing a schistosome vaccine that blocks or impairs parasite migration and the blood-feeding process, it is important to target products of the initial infective stages (*e.g.* SmVAL4, which is likely to be localized in acetabular glands) as well as of established adults (*e.g.* SmVAL7, which is localized in the esophageal gland of adult worms) (Rofatto *et al.*, 2012 ▶).

SmVALs are characterized by a single CAP domain otherwise referred to as SCP/TAPS, NCBI domain cd00168 or Pfam PF00188, which is found in diverse unrelated proteins from bacteria, plants, animals and viruses (Geer *et al.*, 2002 ▶; Gibbs *et al.*, 2008 ▶; Milne *et al.*, 2003 ▶; Cantacessi *et al.*, 2009 ▶; Ding *et al.*, 2000 ▶; Hawdon *et al.*, 1999 ▶; Zhan *et al.*, 2003 ▶; Gao *et al.*, 2001 ▶). The SCP/TAPS domain is a 15 kDa domain that has been implicated in conditions requiring cellular defense or proliferation, including plant responses to pathogens and human brain tumor growth (Ding *et al.*, 2000 ▶; Hawdon *et al.*, 1999 ▶; Zhan *et al.*, 2003 ▶; Gao *et al.*, 2001 ▶; Gibbs *et al.*, 2008 ▶). In addition, some CAP superfamily members have been implicated in binding lipids. The structural characterization of tablysin-15 from a blood-feeding arthropod revealed a hydrophobic channel that binds leukotrienes with submicromolar affinities, indicating that the protein functions as an anti-inflammatory scavenger of eicosanoids (Xu *et al.*, 2012 ▶). The mammalian CAP protein GLIPR2/GAPR-1 is highly overexpressed in glioblastoma multiforme, and binds to the surface of liposomes containing negatively charged lipids (van Galen *et al.*, 2010 ▶, 2012 ▶). GAPR-1 can bind as many as three phosphatidylinositol molecules strongly enough to resist denaturation or organic solvent extraction (van Galen *et al.*, 2010 ▶, 2012 ▶). Additionally, SCP/TAPS family members in yeast have recently been shown to be required for the export of sterols *in vivo* and these proteins bind cholesterol *in vitro* (Choudhary & Schneiter, 2012 ▶). Several structures of SCP/TAPS proteins have been reported and despite low sequence homology they all reveal a conserved α/β-sandwich CAP domain (Asojo *et al.*, 2005 ▶, 2011 ▶; Asojo, 2011 ▶; Serrano *et al.*, 2004 ▶; Fernández *et al.*, 1997 ▶; Wang *et al.*, 2005 ▶; Shikamoto *et al.*, 2005 ▶; Guo *et al.*, 2005 ▶; Xu *et al.*, 2012 ▶; Gibbs *et al.*, 2008 ▶; Borloo *et al.*, 2013 ▶). The main structural differences in the CAP domain include the lengths of their strands and helices as well as the lengths, orientations and locations of loops (Asojo *et al.*, 2011 ▶; Asojo, 2011 ▶). Additionally, previous analysis of CAP protein structures revealed that each protein had long flexible loops that resulted in difficulty in accurately predicting the structures of these proteins (Asojo *et al.*, 2005 ▶, 2011 ▶; Asojo, 2011 ▶). The structural studies of SmVALs were initiated as part of ongoing efforts to characterize the structure and functions of these putative vaccine candidates. Thus, the first crystal structure of a SCP/TAPS or CAP protein from *S. mansoni*, SmVAL4, is presented.

## 2. Experimental procedures   

### 2.1. Recombinant protein expression and purification   

The pPICZαA vector containing the ORF for the mature SmVAL4 protein was constructed as described previously (Farias *et al.*, 2012 ▶), with the inclusion of a stop codon at the end of the mature protein sequence. The transformants in *Pichia pastoris* strain X33 were selected on zeocin-resistant YPD plates and identified by PCR amplification using pPICZαA vector flanking primers (α-factor and 3′AOX1). 20 colonies with the correct insert were picked and screened for induction of recombinant SmVAL4 protein with 0.5% methanol at 30°C for 72 h. The highest expressing colony was used to make research seed stock, from which a starter culture was grown (29°C, 250 rev min^−1^) in 25 ml buffered minimal glycerol (BMGY; 100 m*M* potassium phosphate pH 6.0, 1.34% yeast nitrogen base, 4 × 10^−5^% biotin, 1% yeast extract, 1% glycerol) in a 250 ml baffled flask at 29°C, 300 rev min^−1^ until the OD_600_ reached 2.0 (approximately 18 h). The starter culture was inoculated into 300 ml BMG in a 2.0 l baffled flask and growth was continued until an OD_600_ of between 2 and 6 was reached. The cells were gently pelleted (3000*g*, 5 min at room temperature) and resuspended in 1000 ml buffered minimal methanol (BMMY; 100 m*M* potassium phosphate pH 6.0, 1.34% yeast nitrogen base, 4 × 10^−5^% biotin, 1% yeast extract, 0.5% methanol) to initiate induction. Every 24 h, 100% methanol was added to a final concentration of 0.5% to maintain induction. After 72 h of induction, the culture medium containing the secreted SmVAL4 protein (CSP) was separated from the yeast cells by centrifugation at 10 000 rev min^−1^. The CSP was filtered through a 0.22 µm membrane filter and stored at −80°C until use. After thawing the CSP, 500 ml of the CSP was dialyzed overnight at 4°C against 0.1 *M* Tris–HCl pH 8.0 with a regenerated cellulose membrane with 3.5 kDa molecular-weight cutoff (Fisher Scientific). The extensive dialysis removed the medium and other salts prior to purification. The protein was purified in 150 ml batches by cation-exchange chromatography under native conditions using two 5 ml HiTrap SP XL columns (GE Healthcare) connected in series pre-equilibrated with seven column volumes (CVs) of 0.1 *M* Tris–HCl pH 8.0 and eluted with a 0–1 *M* NaCl linear gradient. Fractions encompassing the main peak and the purity of the preparation were assessed by electrophoresis using a NuPAGE Novex 4–12% Bis-Tris Gel with MES running buffer (Invitrogen). The typical yield from a 1 l shake-flask growth was approximately 25 mg of >99% pure protein.

### 2.2. Crystallization   

The peak fractions were combined and concentrated to 8 mg ml^−1^ in sodium citrate pH 5.0. Crystallization conditions were identified and optimized after screening for crystals using The Cryos and PEGs Suites (Qiagen) and Crystal Screen (Hampton Research). The best crystals were obtained at 298 K by vapor diffusion in sitting drops by mixing 1.5 µl protein solution with an equal volume of reservoir solution consisting of 0.085 *M* sodium acetate trihydrate pH 4.6, 25%(*w*/*v*) PEG 2000, 0.17 *M* ammonium sulfate, 15%(*v*/*v*) glycerol. The crystals were typically shaped like swords and were of dimensions 0.1 × 0.3 × 0.7 mm. We cut each crystal with micro-tools to give a smaller fragment that was used for data collection. Since the crystals grew in solutions that contained adequate cryoprotectant, all crystals were flash-cooled directly in a stream of N_2_ gas at 113 K prior to collecting diffraction data.

### 2.3. Data collection and structure determination   

X-ray diffraction data were collected at the Baylor College of Medicine core facility using a Rigaku HTC detector. The X-ray source was a Rigaku FR-E+ SuperBright microfocus rotating-anode generator with VariMax HF optics. A data set was collected from a single crystal with a crystal-to-detector distance of 105 mm and exposure times of 120 s for 0.5° oscillations using the *CrystalClear* (d*TREK) package (Pflugrath, 1999 ▶). Data were processed using *MOSFLM* (Leslie, 2006 ▶). The crystal belonged to the tetragonal space group *P*6_1_, with approximate unit-cell parameters *a* = 78.65, *b* = 78.65, *c* = 83.52 Å, α = β = 90, γ = 120°.

As was the case with the other SCP/TAP protein structure solved in our laboratory, several attempts at molecular replacement (MR) were performed using different search models (Asojo *et al.*, 2011 ▶, 2005 ▶; Asojo, 2011 ▶) with *Phaser* (McCoy *et al.*, 2005 ▶; Storoni *et al.*, 2004 ▶). The only MR search model that resulted in a solution with an *R*_free_ of less than 40% was sGLIPR1 (PDB entry 3q2r; Asojo *et al.*, 2011 ▶) with major loops removed. The correct MR solution implied a monomer of SmVAL4 per asymmetric unit with a Matthews coefficient (Kantardjieff & Rupp, 2003 ▶; Matthews, 1968 ▶) of 3.97 Å^3^ Da^−1^ and a solvent content of 69%. The model was improved through automatic model building with *ARP*/*wARP* (Morris *et al.*, 2003 ▶, 2004 ▶) followed by manual model-building cycles using *Coot* (Emsley *et al.*, 2010 ▶) and structure refinement with *REFMAC*5 (Murshudov *et al.*, 2011 ▶) within the *CCP*4 package (Winn *et al.*, 2011 ▶). Unless otherwise noted, figures were generated using *PyMOL* (DeLano, 2002 ▶). Details of the quality of the structure as well as data collection are shown in Table 1 ▶. The atomic coordinates and structure factors have been deposited in the PDB as entry 4p27.

### 2.4. Size-exclusion chromatography and multi-angle light scattering (SEC-MALS)   

SEC-MALS experiments were performed by loading 10 µg protein sample onto a TSKgel SuperSW mAb HTP column (Tosoh Biosciences, King of Prussia, Pennsylvania, USA) at a flow rate of 0.25 ml min^−1^ using an Agilent 1260 Infinity series HPLC. The column buffer was 1× PBS (pH 7.4). A UV detector (Agilent), a miniDAWN triple-angle light-scattering detector (Wyatt Technology) and an Optilab rEX Differential Refractometer (Wyatt Technology) were connected in series downstream from the column. The refractometer provided a continuous index of protein concentration. A d*n*/d*c* (refractive-index increment) value of 0.185 ml mg^−1^ was used. Bovine serum albumin was used as an isotropic scatterer for detector normalization. The light scattered by a protein is directly proportional to its weight-average molecular mass and concentration. Therefore, molecular masses were calculated from the light-scattering and interferometric refractometer data using the *ASTRA* 6.0 software.

### 2.5. *In vivo* sterol export from mutant yeast cells   

Acetylation and export of sterols into the culture supernatant was examined as described by Tiwari *et al.* (2007 ▶). Heme (*hem1*Δ) deficient yeast cells were cultivated in the presence of cholesterol/Tween 80-containing medium and were labeled with 0.025 µCi ml^−1^ [^14^C]-cholesterol (American Radiolabeled Chemicals Inc., St Louis, Missouri, USA). Cells were harvested by centrifugation, washed twice with synthetic complete (SC) media, diluted to an OD_600_ of 1 in fresh medium containing non-radiolabeled cholesterol and grown overnight. Cells were centrifuged and lipids were extracted from the cell pellet and the culture supernatant using chloroform/methanol [1:1(*v*/*v*)]. Samples were dried and separated by thin-layer chromatography using silica gel 60 plates (TLC; Merck, Darmstadt, Germany) using the solvent system petroleum ether/diethyl ether/acetic acid (70:30:2 by volume). Radiolabeled lipids on the TLC were quantified by scanning with a Berthold Tracemaster 40 Automatic TLC Linear Analyzer (Berthold Technologies, Bad Wildbad, Germany). The TLC plates were then exposed to phosphorimager screens and radiolabeled lipids were visualized using a phosphorimager (Bio-Rad Laboratories, Hercules, California, USA).

### 2.6. *In vitro* sterol binding   

The radioligand-binding assay was performed as described previously (Im *et al.*, 2005 ▶; Choudhary & Schneiter, 2012 ▶). Purified protein (0–300 pmol) in binding buffer (20 m*M* Tris pH 6.5, 30 m*M* NaCl, 0.05% Triton X-100) was incubated with [^3^H]-cholesterol (50 or 200 pmol) for 1 h at 30°C. The protein was then separated from the unbound ligand by adsorption to Q Sepharose beads (GE Healthcare, USA), the beads were washed and the radioligand was quantified by scintillation counting. For competitive binding assays, unlabeled cholesterol (50 or 500 pmol) was included in the binding reaction. To determine nonspecific binding the ion-exchange beads were incubated in the absence of added protein.

## 3. Results   

### 3.1. Solution structure of SmVAL4   

**Figure 1:**
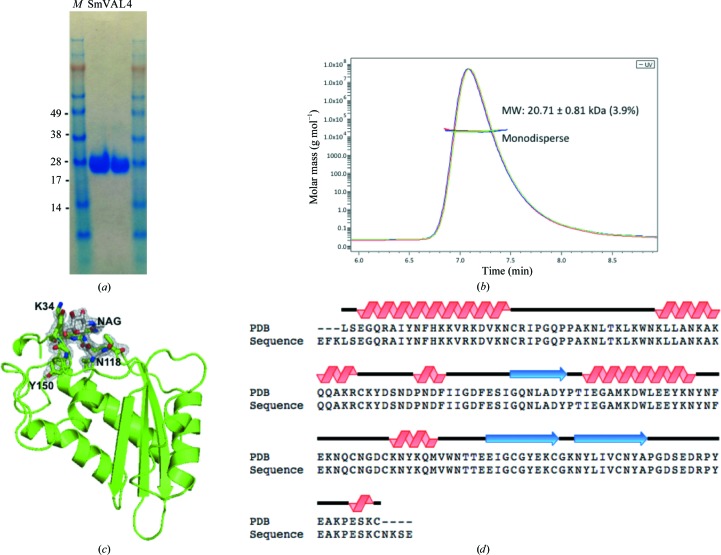
The SmVAL4 monomer. (*a*) A Coomassie-stained SDS gel reveals the purity of the SmVAL4 sample and its monomeric mass of ∼20 kDa. Lane *M* contains molecular-weight marker (labeled in kDa). (*b*) SEC-MALS analysis reveals that SmVAL4 is an ∼20 kDa monomer in solution. (*c*) Fit of *N*-­glycosylated Asn118 and proximal residues into a 2*F*_o_ − *F*_c_ electron-density map (gray) calculated from the refined model of SmVAL4 and contoured at 1.2σ. NAG, *N*-acetyl-d-glucosamine. (*d*) Topology of the SmVAL4 structure.

In order to determine the oligomeric state of recombinant SmVAL4 in solution, the absolute molecular mass of SmVAL4 was measured by size-exclusion chromatography and multi-angle laser light scattering (SEC-MALS). The protein gave a single peak on the sizing column (Fig. 1 ▶ *b*). The light scattered by a protein is directly proportional to its weight-average molecular mass and its concentration. The molecular mass was determined to be 20.71 ± 0.81 kDa, which is consistent with its electrophoretic mobility (Fig. 1 ▶ *a*). The theoretical molecular mass is 18.81 kDa. The difference between the theoretical and experimental molecular mass is likely to be owing to glycosylation of the protein during production in *P. pastoris*. The results indicate that SmVAL4 forms a monomer in solution, unlike several of the previously reported SCP/TAPS or CAP proteins, including GAPR-1 and NaASP2, which formed dimers in solution (Asojo *et al.*, 2005 ▶; Gibbs *et al.*, 2008 ▶).

### 3.2. Overall structure of SmVAL4   

**Figure 2:**
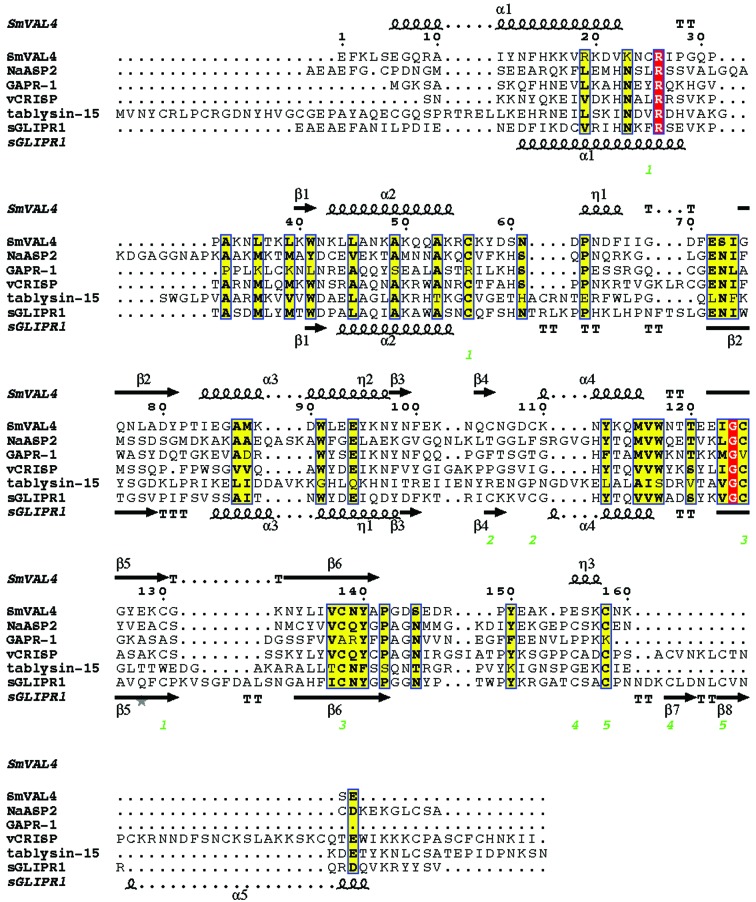
Structural features of SmVAL4 and primary-sequence alignment with selected representative CAP proteins. The sequences were aligned with *ClustalW*2 and the secondary-structural features are illustrated with the coordinates of SmVAL4 and GLIPR2 using *ESPript* (Gouet *et al.*, 2003 ▶). The different secondary-structure elements shown are α-helices (large squiggles labeled α), 3_10_-helices (small squiggles labeled η), β-strands (arrows labeled β) and β-turns (labeled TT). Identical residues are shown in white on a red background and conserved residues are highlighted in yellow. The locations of the cysteine residues involved in disulfide bonds are numbered in green and the signature CRISP motifs are identified by red bars. The representative CAP structures are NaASP2 (PDB entry 1u53), tablysin-15 (PDB entry 3u3n), GAPR-1 (PDB entry 1smb), sGLIPR1 (PDB entry 3qnx) and vCRISP (PDB entry 1rc9).

**Figure 3:**
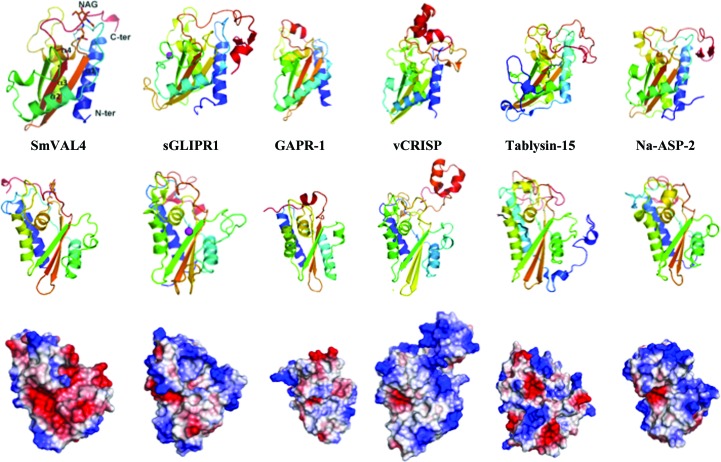
Comparison of the SmVAL4 structure with representative CAP structures. The top row shows ribbon diagrams of SmVAL4 (PDB entry 4p27), NaASP2 (PDB entry 1u53), tablysin-15 (PDB entry 3u3n), GAPR-1 (PDB entry 1smb), sGLIPR1 (PDB entry 3q2u) and vCRISP (PDB entry 1rc9). The core α–β–α sandwich is formed by the three-stranded β-sheet between the labeled helices. The second row is another view of the proteins in which the central cavity is visible. The third row is from the same view as the second row and shows the differences in the charge distribution of these CAP proteins, colored blue for positively charged and red for negatively charged regions. The differences in the charge distribution in proximity to the central cavity is obvious in the structures. The Zn^2+^ complexed with sGLIPR1 and sitting in the central cavity is shown in magenta. The palmitate bound to the tablysin-15 structure is shown in a black stick representation.

The refined model has one monomer of SmVAL4 in the asymmetric unit. The crystallographic oligomer is thus consistent with the solution structure. There is clear unambiguous density for one Asn-linked glycosylation site, which was modeled as an *N*-acetyl-d-glucosamine covalently linked to Asn118 (Fig. 1 ▶ *c*). Native SmVAL4 from the parasite has been shown to be *N*-glycosylated (Farias *et al.*, 2012 ▶); however, it is not known whether the glycosylation of *P. pastoris*-derived recombinant SmVAL4 is identical to that of parasite-derived SmVAL4. The final refined structure contains a total of 155 amino acids, of which 153 have clear unambiguous main-chain and side-chain electron density at greater than 1.0σ. The loop connecting amino-acid residues 59–62 has partly ordered electron density for both the main chain and side chains, and is the only region in the structure that contains any amino acids with Ramachandran outliers, as well as correlation coefficients of less than 0.87. SmVAL4 folds as an αβα sandwich in which a four-mixed-four-stranded β-sheet is sandwiched between two helical/loop regions (Fig. 1 ▶ *c*). The topology of SmVAL4 aligned with its primary sequence is shown in Fig. 1 ▶(*d*). The structure of SmVAL4 is unique and could not have been predicted from other SCP/TAPS protein structures; this is evident from the unique positions of helices and strands in the structure when compared with other CAP proteins (Fig. 2 ▶). SmVAL4 retains the characteristic α/β sandwich CAP structure and a large central cavity (Fig. 3 ▶). However, the two prosite CAP motifs are not conserved and the putative binding cavity lacks all but one of the signature CAP cavity tetrad amino-acid residues (Figs. 2 ▶ and 4 ▶). SmVAL4 has shorter helices and strands than all other reported SCP/TAPS structures and lacks an N-terminal extension (Figs. 2 ▶ and 3 ▶). SmVAL4 has unique loop regions, including a C-terminal extended loop with a terminal helix (Figs. 2 ▶ and 3 ▶). The shape of the C-terminal loop of SmVAL4 is different from those observed for other CAP proteins (Asojo *et al.*, 2011 ▶; Asojo, 2011 ▶). While we show that SmVAL4 is capable of binding lipids, the electron-density maps do not show any evidence of any bound lipid.

### 3.3. Sterol-binding studies   

**Figure 5:**
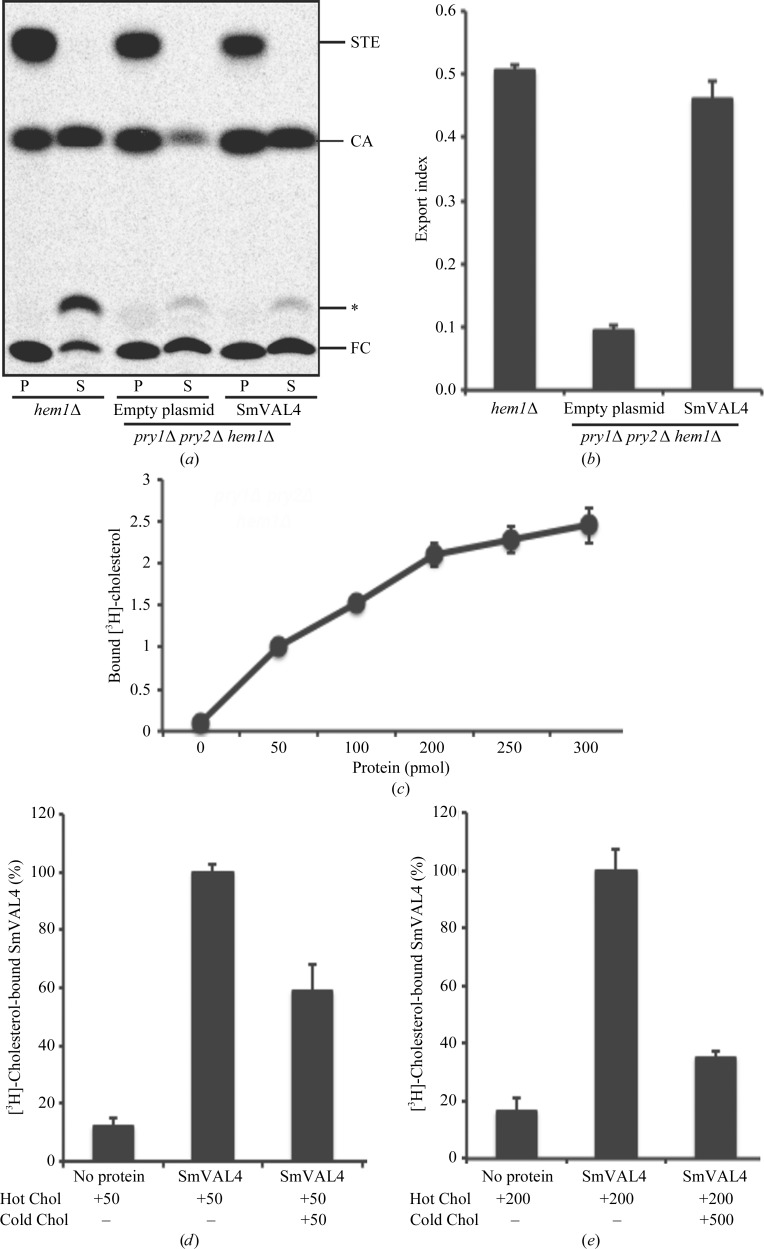
SmVAL4 binds chloresterol *in vivo* and *in vitro*. (*a*) Expression of SmVAL4 complements the sterol-export defect of yeast cells lacking their endogenous CAP proteins. Heme-deficient cells of the indicated genotype containing either an empty plasmid or a plasmid with SmVAL4 were radiolabeled with [^14^C]-cholesterol overnight, washed and diluted in fresh medium to allow export of cholesterol and cholesteryl acetate. Lipids were extracted from the cell pellet (P) and the culture supernatant (S) and separated by thin-layer chromatography. The positions of free cholesterol (FC), cholesteryl acetate (CA) and steryl esters (STE) are indicated on the right. The asterisk marks the position of an unidentified cholesterol derivative. (*b*) Quantification of the export of cholesteryl acetate in yeast cells lacking their endogenous CAP proteins. The export index indicates the relative percentage of cholesteryl acetate that is exported by the cells (the ratio between extracellular cholesteryl acetate and the sum of intracellular and extracellular cholesteryl acetate). Data represent the mean ± SD of two independent experiments. (*c*) SmVAL4 binds cholesterol *in vitro*. Sterol binding was assessed using increasing amounts of the purified protein and 50 pmol [^3^H]-cholesterol as the ligand. The protein was separated from unbound ligand by adsorption to an anion-exchange matrix and bound radioligand was quantified by scintillation counting. (*d*) Addition of an equimolar amount of unlabeled cholesterol (cold Chol) resulted in a corresponding reduction in binding of the radiolabeled ligand (hot Chol). (*e*) Addition of an excess of unlabeled cholesterol reduces binding of the radiolabeled ligand. Purified SmVAL4 (100 pmol) was incubated with 200 pmol [^3^H]-cholesterol as the ligand (hot Chol) and where indicated binding of the radioligand was in competition with 500 pmol unlabeled cholesterol (cold Chol).

The ability of SmVAL4 to bind sterols was tested by expressing it in yeast mutants lacking their endogenous CAP family members Pry1 and Pry2. The yeast Pry proteins bind cholesterol *in vitro* and are required for the export of free cholesterol and cholesteryl acetate into the culture supernatant. To test whether the expression of SmVAL4 in *pry1*Δ *pry2*Δ mutant cells rescued the defect in cholesterol export, heme-deficient cells containing either an empty plasmid or a plasmid with SmVAL4 were radiolabeled with [^14^C]-cholesterol overnight, washed and diluted in fresh medium to allow the export of cholesterol and cholesteryl acetate. Lipids were extracted from the cell pellet (P) and the culture supernatant (S) and separated by thin-layer chromatography (Fig. 5 ▶ *a*). The levels of free cholesterol and cholesteryl acetate were quantified by radio scanning and the relative percentages of cholesteryl acetate that were exported by the cells were plotted as an export index (ratio between extracellular cholesteryl acetate and the sum of intracellular and extracellular cholesteryl acetate; Fig. 5 ▶ *b*). Cells expressing SmVAL4 exported high levels of cholesteryl acetate into the culture supernatant, indicating that SmVAL4 functionally complements the absence of Pry proteins *in vivo*. To determine whether SmVAL4 could bind cholesterol *in vitro*, increasing concentrations of the purified protein (0–300 pmol) were incubated with [^3^H]-cholesterol as a ligand. The protein was separated from unbound ligand by adsorption to an anion-exchange matrix and bound radioligand was quantified by scintillation counting (Fig. 5 ▶ *c*). These experiments revealed a protein concentration-dependent increase in radioligand binding, which indicates that SmVAL4 binds cholesterol *in vitro*. Furthermore, addition of unlabeled cholesterol (50 and 500 pmol) competed with radioligand binding, indicating that chloresterol binding is specific (Figs. 5 ▶ *d* and 5 ▶ *e*). The results of these binding studies indicate that SmVAL4 binds [^3^H]-cholesterol in a concentration-dependent manner and that binding of the radiolabeled ligand can be competed with by incubation with unlabeled cholesterol.

## 4. Discussion   

### 4.1. SmVAL4 is unique and lacks the prototypical CAP motifs   

The structures that were most similar to SmVAL4 were identified using the Structure Similarity option of *PDBeFold* (http://www.ebi.ac.uk/msd-srv/ssm/), which allows a three-dimensional structural alignment that takes both the alignment length and r.m.s.d. into account. The most similar structure to SmVAL4 is that of sGliPR1 with Zn^2+^ (PDB entry 3q2r; Asojo *et al.*, 2011 ▶), indicating that SmVAL4 is more similar to a human CAP protein than to those from other parasites. We had previously observed a similar situation with GSTs from hookworms, which were more structurally similar to human GSTs than to homologues from schistosomes (Asojo *et al.*, 2007 ▶). The structure of NaASP2 (PDB entry 1u53; Asojo *et al.*, 2005 ▶) is the second most similar reported CAP structure to SmVAL4, followed by human GAPR-1 with inositol (PDB entry 4aiw; van Galen *et al.*, 2012 ▶), the GAPR-1 apo structure (PDB entry 1smb; Serrano *et al.*, 2004 ▶) and pseudechetoxin (PDB entry 2dda; Suzuki *et al.*, 2008 ▶), and then by 17 snake-venom CRISP structures and tablysin-15 (PDB entry 3u3n; Xu *et al.*, 2012 ▶). Alignment of these representative CAPs reveals that the greatest differences are in loop regions as well as in the lengths of helices and strands. Despite being a shorter CAP protein than the other representative CAPs, SmVAL4 has longer helices than the other CAP proteins. SmVAL4 also has an additional 3_10_-helix involved in the α–β–α sandwich that was not observed in any of the other representative CAP protein structures (Figs. 2 ▶ and 3 ▶).

Like other CAP or SCP/TAPS structures, SmVAL4 has a large central cavity (Fig. 3 ▶). The CAP cavity is an exposed central cavity observed in all other reported SCP/TAPS protein structures (Asojo, 2011 ▶; Asojo *et al.*, 2005 ▶, 2011 ▶; Gibbs *et al.*, 2008 ▶, ). In many of the SCP/TAPS structures the CAP cavity is typically characterized by residues from the four signature CAP motifs CAP1, CAP2, CAP3 and CAP4 (Gibbs *et al.*, 2008 ▶). Only two of these motifs, CAP1 and CAP2, are defined in the PROSITE database (http://www.expasy.ch/prosite). Interestingly, a PROSITE scan of SmVAL4 reveals that neither of these CAP motifs is conserved. The two additional CAP motifs were defined by Gibbs and coworkers and their consensus sequences are HN*xx*R and G(EQ)N(ILV) for the CAP3 and CAP4 motifs, respectively (Gibbs *et al.*, 2008 ▶). Only the CAP4 motif is present in SmVAL4 and the CAP cavity of SmVAL4 lacks all but one of the signature CAP cavity tetrad amino-acid residues (Figs. 2 ▶ and 4 ▶). Furthermore, none of the histidines that coordinate Zn^2+^ in the proposed Zn^2+^ and heparin-sulfate dependent mechanisms of inflammatory modulation in studies of the cobra CRISP natrin (Wang *et al.*, 2010 ▶) are conserved in SmVAL4 (Figs. 2 ▶ and 4 ▶). The absence of these histidines makes SmVAL4 incapable of coordinating Zn^2+^ and implies different underlying mechanisms for the function of SmVAL4, independent of these residues; indeed, these residues were shown to be unnecessary for sterol transport in yeast CAP proteins (Choudhary *et al.*, 2014 ▶). The lack of both histidines is not unique to SmVAL4; another CAP protein with known structure that lacks both histidines is tablysin-15 (Fig. 2 ▶). The significance of SmVAL4 lacking both histidines remains unknown since several other SmVALs retain both histidines (Chalmers *et al.*, 2008 ▶). Furthermore, based on a comparison of the sequences and structures of group 1 SmVALs it is clear that the structures of most other SmVALs cannot be accurately predicted from the structure of SmVAL4 or those of other representative CAP structures owing to the large number of loop and turn regions in these CAP structures (Supplementary Fig. S1^1^). Additionally, it remains unclear whether any of the SmVALs form the prototypical CAP tetrad motif, and future structural analysis is necessary to determine whether this is the case. It is also unclear how inserted regions and additional terminal regions in many of the SmVALs are folded (Supplementary Fig. S1). The structural characterization of other SmVALs is needed to answer these questions.

### 4.2. An evolutionarily conserved sterol binding function of SmVAL4 and yeast CAP proteins   

**Figure 6:**
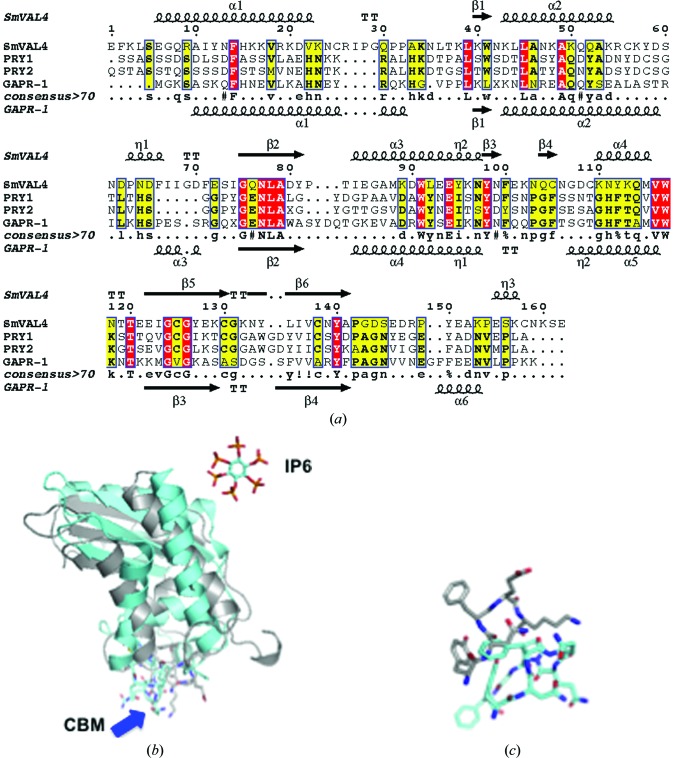
The calveolin-binding motif. (*a*) The conserved calveolin-binding motif (CBM) is evident in the alignment of the sequences of SmVAL4, GAPR1, Pry1 and Pry2. The secondary-structural elements shown are for SmVAL4 and GAPR1 (PDB entry 4aiw). The location of the CBM is identified with a blue line, while the CRISP1 motif is shown as a red line. The figure was generated using *EsPript* and *ClustalW* and structural elements are labeled as described in Fig. 2 ▶. (*b*) The superposed ribbon structures of SmVAL4 (gray) and GAPR1 (cyan) reveals the conformational flexibility of the CBM (shown in stick representation and identified with a blue arrow). The inositol hexakisphosphate (IP6) that was co-crystallized with GAPR1 is shown in a cyan stick representation. (*c*) Close-up of the superposed CBM showing the conformational difference of the loops.

There are three CAP superfamily members in the *Saccharo­myces cerevisiae* genome, Pry1–3, and these proteins are required for cholesteryl acetate transport (Choudhary & Schneiter, 2012 ▶; Choudhary *et al.*, 2014 ▶). A comparison of the sequences reveals that the CAP domain of SmVAL4 shares 35% sequence identity with Pry3 and 28% with both Pry1 and Pry2. The Pry proteins have been shown to be secreted sterol-binding proteins (Choudhary & Schneiter, 2012 ▶) and we showed that SmVAL4 was able to rescue the sterol-binding function of yeast that lacked the endogenous CAP proteins Pry1 and Pry2 (Fig. 5 ▶). While the structures of GAPR1 and SmVAL4 have been solved, those of the Pry proteins are unknown. A structure-based sequence alignment of the CAP domains Pry1 and Pry2 with the structures of SmVAL4 and GAPR1 reveals that the conserved residues are spread through out the CAP domain and are also found in regions that have structural variation (Fig. 6 ▶ *a*).

Recent studies by the Schneiter group revealed that Pry1 contains a calveolin-binding motif (CBM) that was shown to be important for both *in vivo* and *in vitro* sterol binding by Pry1 (Choudhary *et al.*, 2014 ▶). The CBM is conserved in the CAP proteins that have been shown to be implicated in the export of sterol *in vivo* and *in vitro*: Pry1, Pry2, GAPR1 and SmVAL4 (Fig. 6 ▶ *a*). The CBM lies in a flexible loop region that has several polar amino-acid residues capable of interacting with lipids. The CBM loop has different conformations in SmVAL4 and GAPR1 (Figs. 6 ▶ *b* and 6 ▶ *c*), and in both structures there are no sterols in proximity to the loop. It is conceivable that the loops are capable of taking on different conformations to allow the binding of different lipids. The CBM loop is located differently on the opposite face from the reported inositol hexakisphosphate (IP6) observed in the structure of GAPR1 (Fig. 6 ▶ *c*). SmVAL4 lacks the prototypical CRISP1 motif but remains capable of sterol transport, which is consistent with mutational studies that revealed that these motifs were not required for *in vivo* and *in vitro* sterol binding by Pry1 (Choudhary *et al.*, 2014 ▶).

### 4.3. A putative palmitate-binding site   

**Figure 7:**
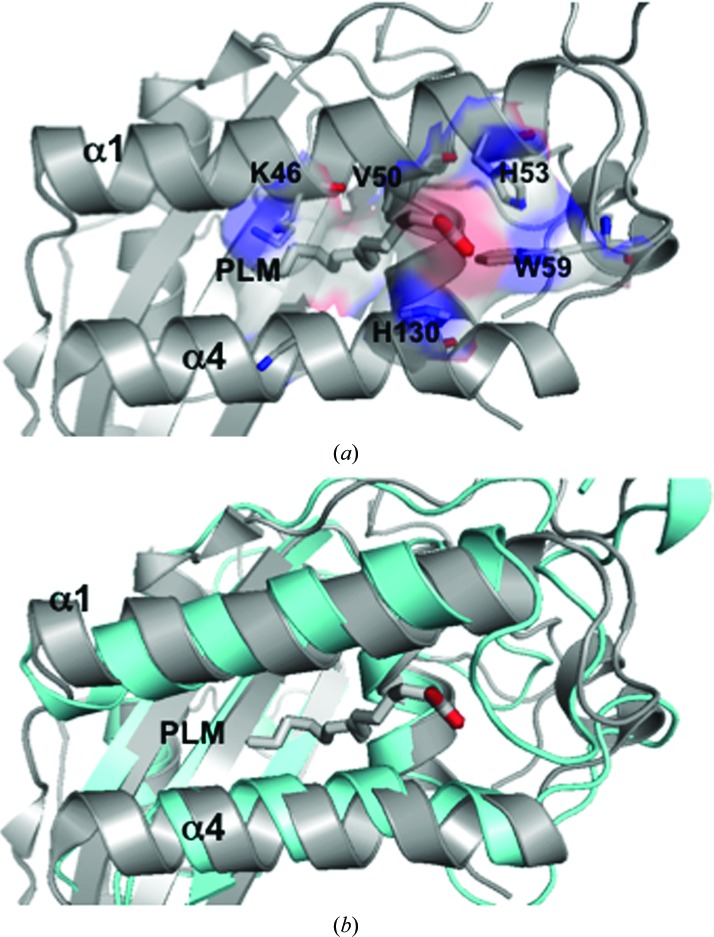
The palmitate-binding motif in tablysin-15. (*a*) Mode of palmitate binding in tablysin-15; the ribbon is shown in gray and interacting residues and palmitate are shown as gray sticks. Also shown is the surface charge in the cavity. (*b*) Superposition of the plamitate-binding region of tablysin-15 (shown in cyan ribbon) with SmVAL4 (shown in gray) reveals that SmVAL4 can have the same palmitate-binding mode as tablysin-15.

The ability of SmVAL4 to bind sterols was confirmed by *in vivo* sterol export from mutant yeast cells, as well as *in vitro* lipid-binding studies. The results of these studies revealed that SmVAL4 binds sterols *in vitro* as well as *in vivo*. Furthermore, the expression of SmVAL4 in yeast cells lacking endogenous CAP function restores the block in sterol export and SmVAL4 binds cholesterol *in vitro*. As part of efforts to correlate sterol binding with structure, we investigated whether any structures of the complex of CAP proteins with sterol-like lipids have been solved. The structure of tablysin-15 in complex with palmitate has previously been reported and the lipid bound in a cavity between two helices, and this cavity was also shown to bind leukotriene (Xu *et al.*, 2012 ▶). A similar cavity was observed between the corresponding α-helices 1 and 4 in the SmVAL4 structure (Figs. 3 ▶ and 7 ▶). The network of residues involved in lipid binding in the tablysin-15 structure includes Lys46, Val50 and His53 from α-helix 1, His130 from α-helix 4 and Trp59 from a flexible loop between α-helix 1 and β-strand 1. Trp59 in tablysin-15 is equivalent to Trp41 in SmVAL4, Lys46 in tablysin-15 is equivalent to Asp21 in SmVAL4, Val50 in tablysin-15 is equivalent to Cys25 in SmVAL4, and His130 in tablysin-15 is equivalent to Asn111 in SmVAL4. While the residues are not conserved across the representative CAPs, there is sufficient space between the equivalent helices to facilitate palmitate binding (Fig. 7 ▶). These analyses reveal that SmVAL4 is structurally able to accommodate lipids such as palmitate, and it is possible that palmitate binding in SmVAL4 is akin to what was observed for tablysin-15. It is important to point out that palmitate binds to tablysin-15 in a different region from the calveolin-binding motif, which suggests at least two lipid-binding regions. Further structural analyses are needed to determine the mode of binding of lipids with SmVAL4, Pry1 and other CAP proteins.

## 5. Conclusions   

The structure of the first SCP/TAPS protein from a schistosome has been refined to 2.16 Å resolution. SmVAL4 is a member of the group 1 VALs of *S. mansoni*. The recombinant protein is *N*-glycosylated and is a monomer in solution, unlike some other reported CAP or SCP/TAPS proteins such as NaASP2, GAPR-1, VesV5 and GLIPR-1, which were shown to form dimers. While SmVAL4 retains the overall α–β–α sandwich characteristic of CAP or SCP/TAPS, it lacks the prototypical CAP tetrad and histidines that are required to coordinate Zn^2+^. The structure of SmVAL4 is unique; it could not have been predicted based on the other CAP structures and it has an unusual C-terminal extension. There is experimental evidence showing that SmVAL4 binds cholesterol *in vitro* and can complement the *in vivo* sterol-export phenotype of yeast mutants lacking their endogenous CAP proteins. The expression of SmVAL4 by yeast cells lacking endogenous CAP function restores the block in sterol export. These studies suggest an evolutionarily conserved lipid-binding function for CAP or SCP/TAPS proteins, including SmVAL4 and yeast Pry1.

## Supplementary Material

PDB reference: SmVAL4, 4p27

Supplementary Figure 1.. DOI: 10.1107/S1399004714013315/be5268sup1.pdf

## Figures and Tables

**Figure 4:**
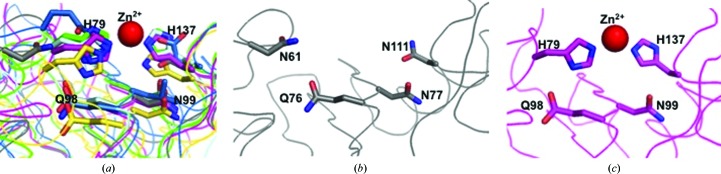
The CAP cavity. (*a*) The superposed central cavity of CAPs reveals that key residues corresponding to the Zn^2+^-binding site superimpose well in representative CAP structures. CAP structures are colored as follows: SmVAL4, gray; NaASP2, green; tablysin-15, blue; GAPR-1, yellow; sGLIPR1, magenta; vCRISP, cyan. The numbers correspond to those for GLIPR1 and the Zn^2+^ ion is shown as a red sphere. (*b*) The same region and view for SmVAL4 alone reveals the absence of the His that coordinates Zn^2+^; numbering corresponds to that of SmVAL4. (*c*) The same region and view for sGLIPR1 alone; numbering corresponds to that of sGLIPR1.

**Table 1:** Statistics of data collection and model refinement for SmVAL4 (PDB entry 4p27) Values in parentheses are for the outer shell.

Space group	*P*6_1_
Unit-cell parameters (Å, °)	*a* = 78.65, *b* = 78.65, *c* = 83.52, α = β = 90, γ = 120
Resolution limits (Å)	30.8–2.16 (2.23–2.16)
〈*I*/σ(*I*)〉	5.1 (1.0)
No. of reflections	342026 (28776)
No. of unique reflections	15770 (1361)
Multiplicity	21.2 (21.1)
*R*_merge_ † (%)	7.1 (76.2)
Completeness (%)	99.7 (99.3)
*R*_cryst_ ‡	0.175 (0.218)
*R*_free_ §	0.208 (0.284)
Correlation coefficient	
*F*_o_ − *F*_c_	0.960
*F*_o_ − *F*_c_ (free)	0.945
R.m.s. deviations	
Bond lengths (Å)	0.024
Bond angles (°)	2.303
Mean *B* factor (Å^2^)	39.37
Model composition	
Monomers	1
Amino-acid residues	155
Water molecules	93
*N*-Acetyl-D-glucosamine	1

† *R*_merge_ = 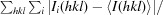 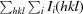, where *I*_*i*_(*hkl*) and 〈*I*(*hkl*)〉 are the *i*th measurement and the mean intensity of the reflection with indices *hkl*, respectively.

‡ *R*_cryst_ = 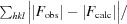 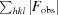, where *F*_obs_ are observed and *F*_calc_ are calculated structure-factor amplitudes.

§ *R*_free_ was calculated using a randomly chosen 5% of reflections.

## Footnotes

1 Supporting information has been deposited in the IUCr electronic archive (Reference: BE5268).
