# *Schistosoma mansoni* venom allergen-like protein 4 (SmVAL4) is a novel lipid-binding SCP/TAPS protein that lacks the prototypical CAP motifs. Corrigendum

**DOI:** 10.1107/S1399004715003132

**Published:** 2015-03-27

**Authors:** Alan Kelleher, Rabih Darwiche, Wanderson C. Rezende, Leonardo P. Farias, Luciana C. C. Leite, Roger Schneiter, Oluwatoyin A. Asojo

**Affiliations:** a National School of Tropical Medicine, Baylor College of Medicine, Houston, TX 77030, USA; b Division of Biochemistry, Department of Biology, University of Fribourg, Chemin du Musée 10, CH 1700 Fribourg, Switzerland; c Centro de Biotecnologia, Instituto Butantan, São Paulo, SP, Brazil

**Keywords:** venom allergen-like protein, Ancylostoma secreted protein, *Schistosoma mansoni*, sperm-coating protein, TAPs, CAP, venom antigen 5, *Saccharomyces cerevisiae*, sterol binding, corrigendum

## Abstract

Figure 6(*a*) of the article by Kelleher *et al.* [(2014), *Acta Cryst.* D**70**, 2186–2196] is corrected.

## Abstract 2

A correction is made to the article by Kelleher *et al.* [(2014), *Acta Cryst.* D**70**, 2186–2196].

In the article by Kelleher *et al.* (2014 ▶), Fig. 6(*a* ▶) was incorrect and the locations of the CBM and Crisp1 were missing. The correct Fig. 6(*a*) is published here and the CBM and Crisp1 are now shown with a blue line and a red line, respectively.

## Figures and Tables

**Figure 6:**
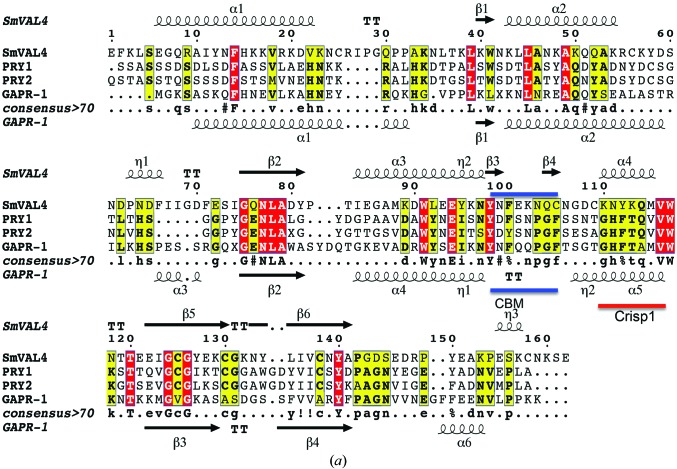
The calveolin-binding motif. (*a*) The conserved calveolin-binding motif (CBM) is evident in the alignment of the sequences of SmVAL4, GAPR1, Pry1 and Pry2. The secondary-structural elements shown are for SmVAL4 and GAPR1 (PDB entry 4aiw). The location of the CBM is identified with a blue line, while the CRISP1 motif is shown as a red line.
