# Supplementary material for: Schistosoma mansoni venom allergen-like protein 4 (SmVAL4) is a novel lipid-binding SCP/TAPS protein that lacks the prototypical CAP motifs
Source: Acta Crystallogr D Biol Crystallogr. 2014 Jul 25;70(Pt 8):2186–96. doi: 10.1107/S1399004714013315 (PMC4118828; doi:10.1107/S1399004714013315)
Supplement: Supplementary file 1 [file d-70-02186-sup1.pdf]

Acta Crystallographica Section D

Volume 70 (2014)

Supporting information for article:

Schistosoma mansoni venom allergen-like protein 4 (SmVAL4) is a novel lipid-binding SCP/TAPS that lacks the prototypical CAP motifs

Alan Kelleher, Rabih Darwiche, Wanderson C. Rezende, Leonardo P. Farias, Luciana C. C. Leite, Roger Schneiter and Oluwatoyin A. Asojo

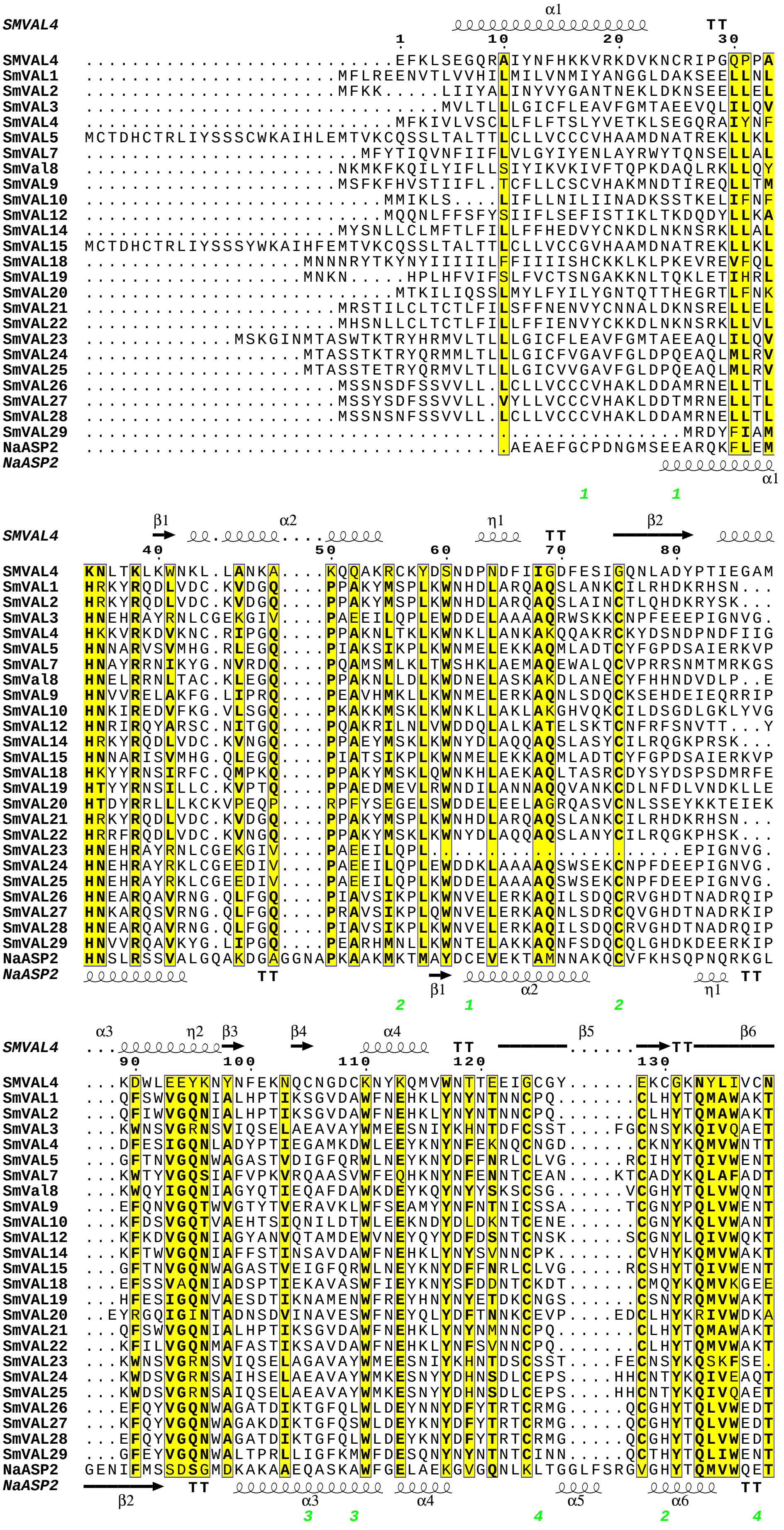

SMVAL4 α1 TT
1 10 20 30
SMVAL4 .........................EFKLSEGQRAIYNFHKKVRKDVKNCRIPGQPPA
SmVAL1 ....................MFLREENVTLVVHILMILVNMIYANGGLDAKSEELLNL
SmVAL2 ....MFKK.....LIIYALINYVYGANTNEKLDKNSEELLEL
SmVAL3 ...........................MVLTLLLGICFLEAVFGMTAEEVQLILQV
SmVAL4 .......................MFKIVLVSCLLFLFTSLYVETKLSEGQRAIYNF
SmVAL5 MCTDHCTRLIYSSSCWKAIHLEMTVKCQSSLTALTTLCLLVCCCVHAAMDNATREKLLKL
SmVAL7 ......................MFYTIQVNFIIFLVLGYIYENLAYRWYTQNSELLAL
SmVal8 ....................NKMKFKQILYIFLLSIYIKVKIVFTQPKDAQLRKLLQY
SmVAL9 ....................MSFKFHVSTIIFL.TCFLLCSCVHAKMNDTIREQLLTM
SmVAL10 ....MMIKLS....LIFLLNILIINADKSSTKELIFNF
SmVAL12 ......................MQQNLFFSFYSIIFLSEFISTIKLTKDQDYLLKA
SmVAL14 ....................MYSNLLCLMFTLFILLFFHEDVYCNKDLNKNSRKLLAL
SmVAL15 MCTDHCTRLIYSSSYWKAIHFEMTVKCQSSLTALTTLCLLVCCGVHAAMDNATREKLLKL
SmVAL18 .................MNNNRYTKYNYIIIIILFFIIIISHCKKLKLPKEVREVFQL
SmVAL19 ....MNKN.....HPLHFVIFSLFVCTSNGAKKNLTQKLETIHRL
SmVAL20 ..........................MTKILIQSSLMYLFYILYGNTQTTHEGRTLFNK
SmVAL21 ....................MRSTILCLTCTLFILSFFNENVYCNNALDKNSRELLEL
SmVAL22 ....................MHSNLLCLTCTLFILLFFIENVYCKKDLNKNSRKLLVL
SmVAL23 ............MSKGINMTASWTKTRYHRMVLTLLLGICFLEAVFGMTAEEAQLILQV
SmVAL24 .................MTASSTKTRYQRMMLTLLLGICFVGAVFGLDPQEAQLMLRV
SmVAL25 .................MTASSTETRYQRMVLTLLLGICVVGAVFGLDPQEAQLMLRV
SmVAL26 ....................MSSNSDFSSVVLL.LCLLVCCCVHAKLDDAMRNELLTL
SmVAL27 ....................MSSYSDFSSVVLL.VYLLVCCCVHAKLDDTMRNELLTL
SmVAL28 ....................MSSNSNFSSVVLL.LCLLVCCCVHAKLDDAMRNELLTL
SmVAL29 ....MRDYFIAM
NaASP2 ..................................AEAEFGCPDNGMSEEARQKFLEM
NaASP2 α1
1 1

SMVAL4 β1 α2 η1 TT β2
40 50 60 70 80
SMVAL4 KNLTKLKWNKL.LANKA....KQQAKRCKYDSNDPNDFIIGDFESIGQNLADYPTIEGAM
SmVAL1 HRKYRQDLVDC.KVDGQ....PPAKYMSPLKWNHDLARQAQSLANKCILRHDKRHSN...
SmVAL2 HRKYRQDLVDC.KVDGQ....PPAKYMSPLKWNHDLARQAQSLAINCTLQHDKRYSK...
SmVAL3 HNEHRAYRNLCGEKGIV....PAEEILQPLEWDDELAAAAQRWSKKCNPFEEEPIGNVG.
SmVAL4 HKKVRKDVKNC.RIPGQ....PPAKNLTKLKWNKLLANKAKQQAKRCKYDSNDPNDFIIG
SmVAL5 HNNARVSVMHG.RLEGQ....PIAKSIKPLKWNMELEKKAQMLADTCYFGPDSAIERKVP
SmVAL7 HNAYRRNIKYG.NVRDQ....PQAMSMLKLTWSHKLAEMAQEWALQCVPRRSNMTMRKGS
SmVal8 HNELRRNLTAC.KLEGQ....PPAKNLLDLKWDNELASKAKDLANECYFHHNDVDLP..E
SmVAL9 HNVVRELAKFG.LIPRQ....PEAVHMKLLKWNMELERKAQNLSDQCKSEHDEIEQRRIP
SmVAL10 HNKIREDVFKG.VLSGQ....PKAKKMSKLKWNKLLAKLAKGHVQKCILDSGDLGKLYVG
SmVAL12 HNRIRQYARSC.NITGQ....PQAKRILNLVWDDQLALKATELSKTCNFRFSNVTT...Y
SmVAL14 HRKYRQDLVDC.KVNGQ....PPAEYMSKLKWNYDLAQQAQSLASYCILRQGKPRSK...
SmVAL15 HNNARISVMHG.QLEGQ....PIATSIKPLKWNMELEKKAQMLADTCYFGPDSAIERKVP
SmVAL18 HKYYRNSIRFC.QMPKQ....PPAKYMSKLQWNKHLAEKAQLTASRCDYSYDSPSDMRFE
SmVAL19 HTYYRNSILLC.KVPTQ....PPAEDMEVLRWNDILANNAQQVANKCDLNFDLVNDKLLE
SmVAL20 HTDYRRLLLKCKVPEQP....RPFYSEGELSWDDELEELAGRQASVCNLSSEYKKTEIEK
SmVAL21 HRKYRQDLVDC.KVDGQ....PPAKYMSPLKWNHDLARQAQSLANKCILRHDKRHSN...
SmVAL22 HRRFRQDLVDC.KVNGQ....PPAKYMSKLKWNYDLAQQAQSLANYCILRQGKPHSK...
SmVAL23 HNEHRAYRNLCGEKGIV....PAEEILQPL.......................EPIGNVG.
SmVAL24 HNEHRAYRKLCGEEDIV....PAEEILQPLEWDDKLAAAAQSWSEKCNPFDEEPIGNVG.
SmVAL25 HNEHRAYRKLCGEEDIV....PAEEILQPLKWDDELAAAAQSWSEKCNPFDEEPIGNVG.
SmVAL26 HNEARQAVRNG.QLFGQ....PIAVSIKPLKWNVELERKAQILSDQCRVGHDTNADRQIP
SmVAL27 HNKARQSVRNG.QLFGQ....PRAVSIKPLQWNVELERKAQNLSDRCQVGHDTNADRKIP
SmVAL28 HNEARQAVRNG.QLFGQ....PIAVSIKPLKWNVELERKAQILSDQCRVGHDTNADRQIP
SmVAL29 HNVVRQAVKYG.LIPGQ....PEARHMNLLKWNTELAKKAQNFSDQCQLGHDKDEERKIP
NaASP2 HNSLRSSVALGQAKDGAGGNAPKAAKMKTMAYDCEVEKTAMNNAKQCVFKHSQPNQRKGL
NaASP2 TT β1 α2 η1 TT
2 1 2

SMVAL4 α3 η2 β3 β4 α4 TT β5 TT β6
90 100 110 120 130
SMVAL4 ...KDWLEEYKNYNFEKNQCNGDCKNYKQMVWNTTEEIGCGY......EKCGKNYLIVCN
SmVAL1 ...QFSWVGQNIALHPTIKSGVDAWFNEHKLYNYNTNNCPQ.......CLHYTQMAWAKT
SmVAL2 ...QFIWVGQNIALHPTIKSGVDAWFNEHKLYNYNTNNCPQ.......CLHYTQMAWAKT
SmVAL3 ...KWNSVGRNSVIQSELAEAVAYWMEESNIYKHNTDFCSST....FGCNSYKQIVQAET
SmVAL4 ...DFESIGQNLADYPTIEGAMKDWLEEYKNYNFEKNQCNGD......CKNYKQMVWNTT
SmVAL5 ...GFTNVGQNWAGASTVDIGFQRWLNEYKNYDFFNRLCLVG.....RCIHYTQIVWENT
SmVAL7 ...KWTYVGQSIAFVPKVRQAASVWFEQHKNYNFENNTCADY....KTCADYKQLAFADT
SmVal8 ...KWQYIGQNIAGYQTIEQAFDAWKDEYKQYNYYSKSCSG.....VCGHYTQLVWQNT
SmVAL9 ...EFQNVGQTWVGTYTVERAVKLWFSEAMYYNFNTNICSSA.....TCGNYPQLVWENT
SmVAL10 ...KFDSVGQTVAEHTSIQNILDTWLEEKNDYDLDKNTCENE......CGNYKQLVWANT
SmVAL12 ...KFKDVGQNIAGYANVQTAMDEWVNEYQYYDFDSNTCNSK.....SCGNYLQIVWQKT
SmVAL14 ...KFTWVGQNIAFFSTINSAVDAWFNEHKLYNYSVNNCPK.......CVHYKQMVWAKT
SmVAL15 ...GFTNVGQNWAGASTVEIGFQRWLNEYKNYDFFNRLCLVG.....RCSHYTQIVWENT
SmVAL18 ...EFSSVAQNIADSPTIEKAVASWFIEYKNYSFDDNTCKDT......CMQYKQMVKGEE
SmVAL19 ...HFESIGQNVAESDTIKNAMENWFREYHNYNYETDKCNGS......CSNYRQMVWAKT
SmVAL20 ..EYRGQIGINTADNSDVINAVESWFNEYQLYDFTNNKCEVP....EDCLHYKRIVWDKA
SmVAL21 ...QFSWVGQNIALHPTIKSGVDAWFNEHKLYNYNMNNCPQ.......CLHYTQMAWAKT
SmVAL22 ...KFILVGQNMAFASTIKSAVDAWFNEHKLYNFSVNNCPQ.......CLHYTQMVWAKT
SmVAL23 ...KWNSVGRNSVIQSELAGAVAYWMEESNIYKHNTDSCSST....FECNSYKQSKFSE.
SmVAL24 ...KWDSVGRNSAIHSELAEAVAYWMKESNYYDHNSDLCEPS....HHCNTYKQIVEAQT
SmVAL25 ...KWDSVGRNSAIQSELAEAVAYWMKESNYYDHNSDLCEPS....HHCNTYKQIVQAET
SmVAL26 ...EFQYVGQNWAGATDIKTGFQLWLDEYNNYDFYTRTCRMG.....QCGHYTQLVWEDT
SmVAL27 ...KFQYVGQNWAGAKDIKTGFQSWLDEYKNYDFYTRTCRMG.....QCGHYTQLVWEDT
SmVAL28 ...EFQYVGQNWAGATDIKTGFQLWLDEYKNYDFYTRTCRMG.....QCGHYTQLVWEDT
SmVAL29 ...GFEYVGQNWALTPRLLIGFKMWFDESQNYNYNTNTCINN.....QCTHYTQLIWENT
NaASP2 GENIFMSSDSGMDKAKAAEQASKAWFGELAEKGVGQNLKLTGGLFSRGVGHYTQMVWQET
NaASP2 β2 TT α3 α4 α5 α6 TT
3 3 4 2 4

```
                                 η3
SMVAL4    →
      140        150                160
SMVAL4    YAPGDSEDRPYE......AKPESKCNKSE...............................
SmVAL1    TDIGCGVANCPR.....YGLSIVCNYGPGGNWNNEKPYEMKPRNLCPKVQNIPKNSLQTN
SmVAL2    TDIGCGVANCPR.....YGLSIVCNYGPGGNFNNEKPYEVKPRNMCPKVQNIPKNSLQTS
SmVAL3    AYVGCGFTRCRQY.EYPSSMLIACYYSPKVKSGPPYTDATNRRCKYLNANLNKIKGKGK.
SmVAL4    EEIGCGYEKCGK......NYLIVCNYAPGDS..EDRPYEAKPESKCNKSE..........
SmVAL5    TDIGCGVATCPH...SPFKLSIVCNYGPGGGCPRQFPYSVKGLYRQWTIKYGRKWYSWRY
SmVAL7    THIGCGYAMCFNLTGLDK.VFVVCNYGPGGKYANRQPYDPIYPEDPYYLP..........
SmVal8    THVGCGITNCTGSYSFPYGLSVVCNYGPGGNYEGRYPYEAKSQDECYATTTKRPTTTKRP
SmVAL9    TDVGCGVTDCPN...FRTKLVIVCNYGPGG......NIPEQRPYRTA.............
SmVAL10   TDIGCASNKCGN......RYMVVCNYAPGAD..DERPYEKDS..................
SmVAL12   THIGCGVTDCRKSPQFPYGVFVVCNYAPGAKFD.KSPYDVVSHAKCSILELK........
SmVAL14   TDIGCGVANCQR.....YGLSVVCYYGPGGNWINEKPYKVKPHNLCPIVQSIPKDHFQTN
SmVAL15   TDIGCGVATCPH...SPFKLSIVCNYGPGGGCPRQFPYSVKGLYRQWTIKYGRKWYSWRY
SmVAL18   TEIGCGVQKCSNR......FLVVCNYSPAAE.EDKQPYEKGTQENCDDVDDAEY......
SmVAL19   KHIGCGLNKCKKRK.....LMIVCNYSPSAD.DKGKPYVIGDRSQCLLNE..........
SmVAL20   EFIGCSVGHCNTHTEVKSGQIIVCYYSPMGDVKTSQPYVENKIDPCRHPPITTTTTTKPI
SmVAL21   TDIGCGVANCPR.....YGLSIVCNYGPGGNWNNEKPYEVKPRHMCSMMQNIPQNSLQTN
SmVAL22   TDIGCGVANCSM.....YGLSIVCNYGPGGNWINEKPYEVKPHNLCPIVQNISKDSFPTN
SmVAL23   ......FSSVNAH.CFPTSLCLLFYSDSHSDT...CIFCSLRQCMVDHSSL.........
SmVAL24   AYVGCGYTRCEEY.EYPSNMLIACYYSPKVMSGPPYTDGTNGRCGSE.............
SmVAL25   AYVGCGYTRCEEY.EYPSNMLVACYYSPKMMSGSPYTDGTNGRCGSE.............
SmVAL26   TDVGCGVTDCPN...FPYGLSIVCNYGPGG......NYPGRPLYRTT.............
SmVAL27   TDVGCGVTKCPN...FPYGLSIVCNYGPGG......NYAGRPLYRTT.............
SmVAL28   TDVGCGVTDCPN...FPYGLSIVCNYGPGG......NYPGRPLYRTT.............
SmVAL29   TDFGCGVTECKN...MLPNLNVICNYGPG...............................
NaASP2    VKLGCYVEACSN......MCYVVCQYGPAGNMMGKDIYEKGEPCSKCENCDKEKGLCSA.
NaASP2     ──────→               ──────→      TT                 →    →
              β3                    β4                           β5   β6
              3     2               31                 4  5  4      5

SMVAL4

SMVAL4    ............................................................
SmVAL1    SAHTQHGPKPLTQSEERVSTNVQNGRRECNQREQLRSRY.....................
SmVAL2    RAHTQHVQKPIKQSQKKVSWIVQNGRKDRNQRKQSRSRY.....................
SmVAL3    ............................................................
SmVAL4    ............................................................
SmVAL5    GRRCRPVYVKQRCNHTNERQLNRTPAIQKKRYKLIKQYLCPNKQKV..............
SmVAL7    ............................................................
SmVal8    TTTKRPSTTTTKRPGTTPTQKPGVPKQIPKPIWPSIISTWNEYATSNMIQGIVTQTCICV
SmVAL9    ............................................................
SmVAL10   ............................................................
SmVAL12   .......................................WKRFNLN.......GQNCYCY
SmVAL14   RVHTRHGQKSINLREKKG...VKYRPK.................................
SmVAL15   GRRCHREYVKQRCNHTNERQLNQTPVIQTKGYKLIKQYLCPNKQKV..............
SmVAL18   ............................................................
SmVAL19   ............................................................
SmVAL20   TVTRKLQMGPYGRYRCDCKCQP......................................
SmVAL21   SAHTQHGQKPIKKSEERVSRNMQYGRRIRDLRVYPKYRD.....................
SmVAL22   RAHTRRGQKSLKQIEKKG...VKYHSN.................................
SmVAL23   ............................................................
SmVAL24   ............................................................
SmVAL25   ............................................................
SmVAL26   ............................................................
SmVAL27   ............................................................
SmVAL28   ............................................................
SmVAL29   ............................................................
NaASP2    ............................................................
NaASP2

SMVAL4

SMVAL4    .
SmVAL1    .
SmVAL2    .
SmVAL3    .
SmVAL4    .
SmVAL5    .
SmVAL7    .
SmVal8    K
SmVAL9    .
SmVAL10   .
SmVAL12   L
SmVAL14   .
SmVAL15   .
SmVAL18   .
SmVAL19   .
SmVAL20   .
SmVAL21   .
SmVAL22   .
SmVAL23   .
SmVAL24   .
SmVAL25   .
SmVAL26   .
SmVAL27   .
SmVAL28   .
SmVAL29   .
NaASP2    .
NaASP2
```

Figure S.1. Comparison of the group 1 SmVALs. The group 1 SmVALs were aligned with clustalW2 and the illustrated secondary structural features are of the coordinates of SmVAL4 and NaASP2 (pdb entry 1u53). The alignment reveals that neither SmVAL4 nor NAASP2 can be accurately used to predict the structures of all the group1 SmVALs. Additionally, CAP tetrad, shown in arrow is conserved in some of the SmVALs. As with figure 2, this figure was generated with ESPript (Gouet *et al.*, 2003). The different secondary structure elements shown are alpha helices as large squiggles labelled (α), 3_{10}-helices as small squiggles labelled (η), beta strands as arrows (β), and beta turns (TT). Identical residues are shown in solid red, and conserved residues are highlighted yellow. The locations of the cysteine residues involved in disulfide bonds are numbered in green

The 29 *S. mansoni* SmVALs are the following: SmVAL1 (AAY43180.1), SmVAL2 (XP_002571733.1), SmVAL3 (AAZ04923.2), SmVAL4 (XP_002571676.1), SmVAL5 (ABB88846.2), SmVAL6 (AAY28955.1), SmVAL7 (AAZ04924.1), SmVAL8 (ABW98681.1), SmVAL9 (XP_002582201.1), SmVAL10 (ABO09814.2), SmVAL11 (ABA54555.1), SmVAL12 (XP_002571731.1), SmVAL13 (ABB88843.1), SmVAL14 (XP_002569793.1), SmVAL15 (XP_002582174.1), SmVAL16 (XP_002571817.1), SmVAL17 (XP_002578833.1), SmVAL18 (XP_002571658.1), SmVAL19 (XP_002571657.1), SmVAL20 (CAZ28636.1), SmVAL21 (XP_002578075.1), SmVAL22 (XP_002574629.1), SmVAL23 (XP_002582175.1), SmVAL24 (XP_002574962.1), SmVAL25 (XP_002574963.1), SmVAL26 (XP_002577262.1), SmVAL27 (XP_002577271.1), SmVAL28 (XP_002582199.1) and SmVAL29 (XP_002571340.1).
